# High-intensity versus low-intensity noninvasive positive pressure ventilation in patients with acute exacerbation of chronic obstructive pulmonary disease (HAPPEN): study protocol for a multicenter randomized controlled trial

**DOI:** 10.1186/s13063-018-2991-y

**Published:** 2018-11-21

**Authors:** Zujin Luo, Chao Wu, Qi Li, Jian Zhu, Baosen Pang, Yan Shi, Yingmin Ma, Zhixin Cao, The HAPPEN collaboration group, Baosen Pang, Bing Dai, Bing Sun, Chao Wu, Chao Zhang, Chen Liu, Dan Wang, Dawei Zheng, Dongmei Li, Fucheng An, Hangyong He, Heli Wang, Hongmei Wu, Hongru Liu, Huiqing Ge, Jian Zhu, Jing Zhou, Juan Wang, Kailiang Duan, Kailv Wang, Lianxiang Guo, Lin Chen, Ling Yu, Lirong Liang, Lixin Xie, Longguang Wu, Lujiang Li, Manyan Zhang, Mingdong Hu, Mingwei Chen, Panpan Zhang, Pengfei Li, Qi Li, Qian Li, Qiang Dang, Qingyuan Zhan, Rongbo Wang, Rui Sun, Runxia Shao, Sanhong Zhang, Shengyu Wang, Sijie Liu, Tianjun Chen, Wenshuai Feng, Wujie Lu, Xiaohong Yang, Xiaohua Hou, Xiaoping Zhang, Xiaowu Tan, Xinghui Wu, Xiuhong Jing, Xiuhong Ma, Xiuzhi Yang, Yaoming Hu, Yichong Li, Yingmin Ma, Yonghong Xiang, Yongpeng An, Yongxiang Zhang, Yunhui Lv, Yurong Zhang, Zhaohui Tong, Zhifang Liu, Zhijun Feng, Zhixin Cao, Zhiyang Yin, Zujin Luo

**Affiliations:** 1 Department of Respiratory and Critical Care Medicine, Beijing Engineering Research Center of Respiratory and Critical Care Medicine, Beijing Institute of Respiratory Medicine, Beijing Chao-Yang Hospital, Capital Medical University, 5 Jingyuan Road, Shijingshan District, Beijing, 100043 China; 2 Department of Respiratory and Critical Care Medicine, People’s Hospital of Xinjiang Uygur Autonomous Region, No. 91 Tianchi Road, Tianshan District, Urumqi, 830001 China; 3 Department of Respiratory and Critical Care Medicine, Army Institute of Respiratory Disease, Chongqing Xin-Qiao Hospital, Army Military Medical University, 183 Xinqiao Main Street, Shapingba District, Chongqing, 400073 China; 4 School of Automation Science and Electrical Engineering, Beihang University, No. 37 Xueyuan Road, Haidian District, Beijing, 100191 China

**Keywords:** Noninvasive positive pressure ventilation, High-intensity, Low-intensity, Chronic obstructive pulmonary disease, Exacerbation, Hypercapnia, Endotracheal intubation, Mortality

## Abstract

**Background:**

Despite the positive outcomes of the use of noninvasive positive pressure ventilation (NPPV) in patients with acute exacerbation of chronic obstructive pulmonary disease (AECOPD), NPPV fails in approximately 15% of patients with AECOPD, possibly because the inspiratory pressure delivered by conventional low-intensity NPPV is insufficient to improve ventilatory status for these patients. High-intensity NPPV, a novel form that delivers high inspiratory pressure, is believed to more efficiently augment alveolar ventilation than low-intensity NPPV, and it has been shown to improve ventilatory status more than low-intensity NPPV in stable AECOPD patients. Whether the application of high-intensity NPPV has therapeutic advantages over low-intensity NPPV in patients with AECOPD remains to be determined. The high-intensity versus low-intensity NPPV in patients with AECOPD (HAPPEN) study will examine whether high-intensity NPPV is more effective for correcting hypercapnia than low-intensity NPPV, hence reducing the need for intubation and improving survival.

**Methods/design:**

The HAPPEN study is a multicenter, two-arm, single-blind, prospective, randomized controlled trial. In total, 600 AECOPD patients with low to moderate hypercapnic respiratory failure will be included and randomized to receive high-intensity or low-intensity NPPV, with randomization stratified by study center. The primary endpoint is NPPV failure rate, defined as the need for endotracheal intubation and invasive ventilation. Secondary endpoints include the decrement of arterial carbon dioxide tension from baseline to 2 h after randomization, in-hospital and 28-day mortality, and 90-day survival. Patients will be followed up for 90 days after randomization.

**Discussion:**

The HAPPEN study will be the first randomized controlled study to investigate whether high-intensity NPPV better corrects hypercapnia and reduces the need for intubation and mortality in AECOPD patients than low-intensity NPPV. The results will help critical care physicians decide the intensity of NPPV delivery to patients with AECOPD.

**Trial registration:**

ClinicalTrials.gov, NCT02985918. Registered on 7 December 2016.

**Electronic supplementary material:**

The online version of this article (10.1186/s13063-018-2991-y) contains supplementary material, which is available to authorized users.

## Background

Chronic obstructive pulmonary disease (COPD) is a common, preventable, and treatable disease, characterized by persistent respiratory symptoms and limited airflow [1]. Based on the Burden of Obstructive Lung Disease program and other large-scale epidemiological studies, there were 384 million patients with COPD worldwide in 2010, with a global prevalence of 11.7% [1, 2]. Remarkably, COPD is currently the fourth leading cause of death in the world and is projected to be the third leading cause by 2020, resulting in an economic and social burden that is both substantial and increasing [1, 3, 4]. Acute exacerbation of COPD (AECOPD) is defined as an acute worsening of respiratory symptoms resulting in additional therapy, and it is characterized as an acute clinical event that negatively affects health status and hospitalization and readmission rates, and it may even increase the rates of comorbidities and COPD-related mortality [1, 5, 6]. Hence, it is of great importance to investigate effective treatments or enhance current therapeutic measures for improving prognosis.

Over the past two decades, noninvasive positive pressure ventilation (NPPV) has been increasingly used in the care of patients with AECOPD [7–11]. Several lines of evidence strongly support its use in these patients [12]. Numerous previous studies [13–19] have indicated that NPPV corrects ventilatory status, reduces the need for invasive ventilation, and improves prognosis more than conventional oxygen therapy. However, NPPV still fails in approximately 15% of AECOPD patients; in this group, mortality is not reduced [18–20]. There are several possible reasons for this. First, in the conventional approach, in which a relatively lower inspiratory positive airway pressure (IPAP) is used, termed low-intensity NPPV, pH and arterial carbon dioxide tension (PaCO_2_) continuously worsen, and it becomes difficult to recover consciousness in a small proportion of AECOPD patients, in spite of NPPV use [20–22]. Second, although PaCO_2_ can be decreased through the use of conventional NPPV in the majority of AECOPD patients, it is still difficult to normalize ventilatory status, and it can easily worsen if there is a slight change in a patient’s clinical situation, which may trigger NPPV failure [16, 19]. Third, if PaCO_2_ is not decreased to a normal level, it might have a harmful effect on the internal environment and vital organs. Finally, for a minority of AECOPD patients, NPPV is poorly tolerated and must be discontinued, possibly because of the inadequate pressure support provided by low-intensity NPPV; this ultimately requires endotracheal intubation [23, 24].

High-intensity NPPV, a form of pressure-limited ventilation, combined with stepwise titration of IPAP up to 30 cmH_2_O, was introduced as a novel therapeutic option in an attempt to maximally decrease elevated PaCO_2_ to normal levels [25, 26]. In theory, high-intensity NPPV may be more efficient than low-intensity NPPV in augmenting alveolar ventilation and offsetting the extra dead space caused by the face mask, and it can reduce the inspiratory work of breathing and alleviate dyspnea, bringing greater comfort and tolerance. Windisch et al. [27] reported that, in patients with stable hypercapnic COPD, improvements in PaCO_2_, lung function, and breathing pattern were achievable using high-intensity NPPV. Dreher et al. [28], in a study on patients with stable hypercapnic COPD, directly compared high-intensity NPPV (mean IPAP of 29 cmH_2_O) with the conventional approach, which uses a considerably lower IPAP (mean of 15 cmH_2_O), and found that high-intensity NPPV was superior to low-intensity NPPV in controlling nocturnal hypoventilation, thus improving lung function, dyspnea during physical activity, and health-related quality of life. However, to the best of our knowledge, it remains unclear whether high-intensity NPPV is superior to low-intensity NPPV in improving ventilatory status (and thus reducing the need for intubation and mortality rate) in patients with AECOPD.

We hypothesize that high-intensity NPPV will be more effective for correcting hypercapnia than low-intensity NPPV, thereby reducing the need for intubation and improving survival in AECOPD patients. Accordingly, the high-intensity versus low-intensity NPPV in patients with AECOPD (HAPPEN) study will be performed to assess the efficacy of high-intensity NPPV in the care of AECOPD patients in comparison with low-intensity NPPV.

## Methods/design

### Aims

The primary aim of the HAPPEN study is to determine whether high-intensity NPPV is more effective for correcting hypercapnia than low-intensity NPPV in patients with AECOPD. The secondary aim is to determine whether high-intensity NPPV is more effective than low-intensity NPPV for decreasing PaCO_2_ from baseline to 2 h after randomization and hospital mortality, and improving 28-day mortality and 90-day survival.

### Study design

**Fig. 1:**
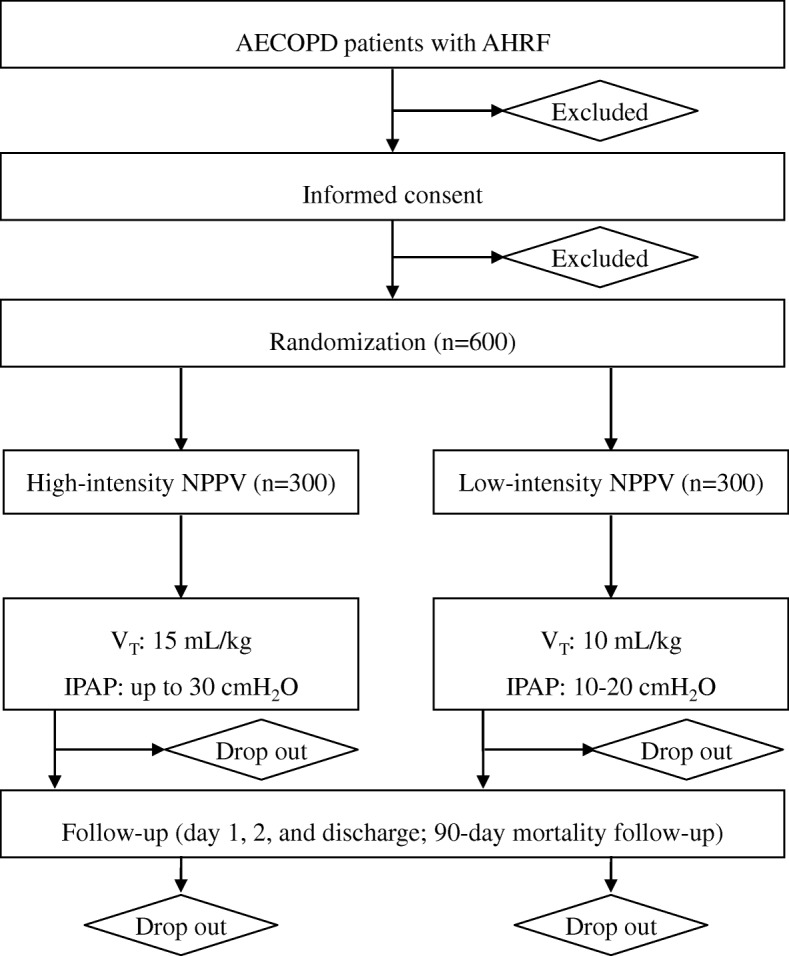
CONSORT diagram. *AECOPD* acute exacerbation of chronic obstructive pulmonary disease, *NPPV* noninvasive positive pressure ventilation, *V*_*T*_ tidal volume, *IPAP* inspiratory positive airway pressure

**Fig. 2:**
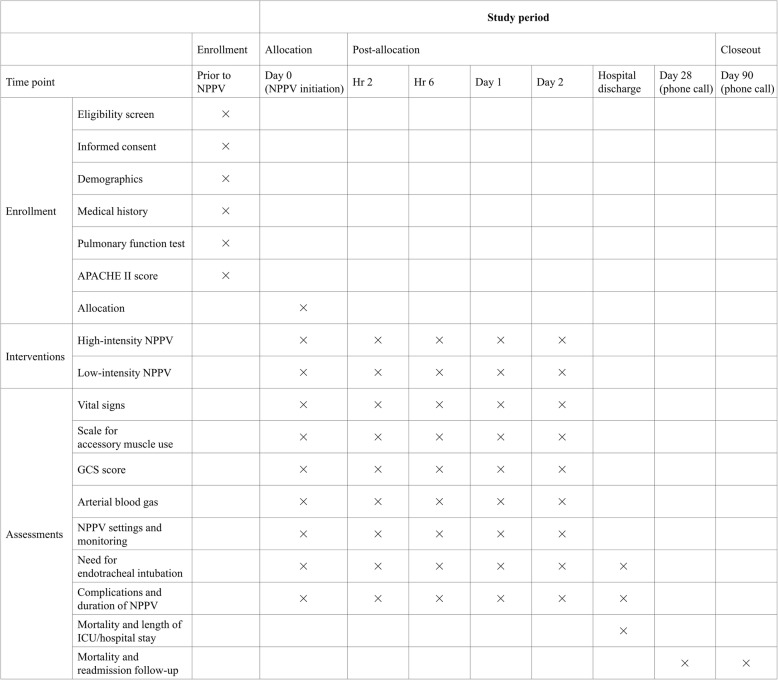
SPIRIT schedule of enrollment, intervention, and assessments. *APACHE* Acute Physiology And Chronic Health Evaluation, *NPPV* noninvasive positive pressure ventilation, *GCS* Glasgow coma scale *ICU* intensive care unit

The HAPPEN study is a multicenter, two-arm, single-blind, prospective, randomized controlled trial initiated by the investigators of the HAPPEN collaboration group. In total, 600 AECOPD patients with low to moderate hypercapnic respiratory failure admitted to a network of 27 respiratory wards from university hospitals in China will be included and randomized to receive high-intensity or low-intensity NPPV. The protocol structure was written in compliance with the Consolidated Standards of Reporting Trials (CONSORT) 2010 statement guidelines, and it follows the Standard Protocol Items: Recommendations for Interventional Trials (SPIRIT) 2013 statement. The CONSORT diagram of the study is shown in Fig. 1, and the SPIRIT schedule for this trial is given in Fig. 2. The complete SPIRIT checklist for the study is provided in Additional file 1.

The trial was designed in accordance with the fundamental principles set forth in the Declaration of Helsinki and the requirements established by Chinese legislation in the field of biomedical research for the protection of personal data and concerning bioethics. The protocol has reached version 6, in which there were no further changes. The approval of the final protocol by the ethics committee at each participating center was obtained before recruitment was initiated. For inclusion in the study, signed written informed consent will be obtained from the patients, patients’ next of kin, or other surrogate decision makers, if appropriate (see Additional file 2). The study was registered on December 7, 2016 at https://ClinicalTrials.gov with identification number NCT02985918.

### Study population

During screening and prior to enrollment in the study, patients will be considered eligible for the study if they are diagnosed with AECOPD as defined by the criteria of the Global Initiative for Chronic Obstructive Lung Disease (GOLD) in 2017, with arterial pH < 7.35 and ≥ 7.25, and PaCO_2_ > 45 mmHg [1].

Patients will be excluded from study participation if any of the following criteria are present: age < 18 years; excessive amount of respiratory secretions or weak cough; upper airway obstruction; recent oral, facial, or cranial trauma or surgery; recent gastric or esophageal surgery; severe abdominal distension; active upper gastrointestinal bleeding; cardiac or respiratory arrest; pH < 7.25; arterial oxygen tension/fraction of inspired oxygen (PaO_2_/FiO_2_) < 150 mmHg; pneumothorax; severe ventricular arrhythmia or myocardial ischemia; severe hemodynamic instability despite fluid repletion and use of vasoactive agents; severe metabolic acidosis; lack of cooperation; and refusal to receive NPPV [29]. Patients will also be excluded if they have a do-not-intubate order or refuse research authorization.

### Recruitment

Investigators are screening consecutive patients with AECOPD every day at the 27 respiratory wards. This screening started in December 2017, and a recruitment time of 12 months has been estimated, based on the estimation of the incidence of AECOPD and patient characteristics at the first author’s hospital. Demographic data for screened patients are being recorded regardless of whether the patients meet the enrollment criteria. All are being reviewed with respect to all inclusion and exclusion criteria.

If patients meet all inclusion criteria and no exclusion criteria, the investigators will explain the nature and the aim of the study to the patients; if patients express an interest in participating, investigators will ask them to sign the informed consent form (see Additional file 2). Patients who meet all inclusion criteria and no exclusion criteria and sign the informed consent form will be included in the study.

### Randomization and blinding

Randomization will be accomplished by a computer-generated random number sequence, stratified by study center, with an allocation ratio of 1:1 for each group. The generation of sequences and their storage will be managed by an independent information technology expert who is not involved in this study. Each allocation sequence will be concealed from the coordinating center through the use of numbered, opaque, and sealed envelopes, and will be managed by an independent employee at the coordinating center who is not involved in this study. The randomization number will consist of a patient identification number of nine digits, in which the first four digits correspond to the identifier of the study center, the middle three correspond to the patient inclusion number at the respective center, and the remaining two correspond to the NPPV-strategy identifier. An independent telephone-contact system will be used for randomization. All investigators at each of the participating centers will immediately contact an independent employee of the coordinating center to obtain a randomization number if a patient fulfills the inclusion criteria. Within 1 h of the validation of the inclusion criteria, patients will be randomly assigned to undergo either high-intensity or low-intensity NPPV. Patients will continue to receive their current therapy prior to randomization assignment.

At each study center, at least two investigators will be conducting the study: one to perform the interventions defined in the protocol, and the other, who will be blinded to the intervention, to perform the data collection. All data related to the NPPV parameters will be extracted from a cloud platform by an independent information technology expert not involved in this study; all data analyses will be performed in a blinded fashion.

### High-intensity NPPV

In the high-intensity NPPV group, patients will undergo pressure-limited NPPV (e.g., NPPV in spontaneous/timed mode) at a higher IPAP level. IPAP is initially set at 10 cmH_2_O and continuously adjusted by increments and decrements of 1–2 cmH_2_O (up to 30 cmH_2_O), according to patients’ tolerance, to obtain a tidal volume (*V*_T_) of 15 mL/kg of predicted body weight (PBW). The PBW is calculated according to a predefined formula: 50 + 0.91 × (centimeters of height − 152.4) for men and 45.5 + 0.91 × (centimeters of height − 152.4) for women. IPAP should be increased as much as possible to decrease PaCO_2_ to a normal level. However, if PaCO_2_ decreases to less than 35 mmHg, IPAP should be decreased to achieve normocapnia.

### Low-intensity NPPV

In the low-intensity NPPV group, patients will undergo pressure-limited NPPV (e.g., NPPV in spontaneous/timed mode) with a conventional IPAP level. IPAP is initially set to 10 cmH_2_O and is continuously adjusted by increments and decrements of 1–2 cmH_2_O (up to 20 cmH_2_O), according to patients’ tolerance, to obtain a *V*_T_ of 10 mL/kg of PBW [13, 15, 19].

### NPPV implementation

NPPV will be performed by investigators with considerable experience. Both high-intensity and low-intensity NPPV will be provided using the same noninvasive ventilator with a cloud platform that can provide data transmission and exchange over a wireless network (Resmart GII Y-30 T, BMC Medical Co., Ltd., Beijing, China). An oro-nasal mask of the proper size (iVolve Full Face Mask BMC-FM, BMC Medical Co.; http://www.bmc-medical.com) will be the first choice, but a nasal mask (N4 Nasal Mask, BMC Medical Co.) may be used if a patient does not tolerate the oro-nasal mask. A disposable single-limb circuit will be used, and a leak port will be incorporated into the mask. Our standard procedures are described as follows.

In both groups, expiratory positive airway pressure (EPAP) is set at 5–8 cmH_2_O, the back-up respiratory rate (RR) is set to 10 breaths/min, rise time is set to 25–200 ms, and the inspiratory time is set at a minimum of 0.5 s and a maximum of 2.0 s. Supplemental oxygen is supplied through a port in the mask, with the flow rate adjusted to maintain an oxygen saturation between 90 and 95%.

All patients lie in a semi-recumbent position, with the head of the bed elevated to an angle of 30–45°. All patients included in the study will be encouraged to use NPPV as much as possible during the first 6 h after randomization and at least 10 h per day, and they will be rigorously monitored at bedside to ensure optimal NPPV use. Disconnection from the ventilator is allowed for short periods (to clear secretions, drink water, or eat food), but it is not scheduled.

A heated humidifier (H60 heated humidifier, BMC Medical Co.) is used to guarantee the delivered gas, with a 100% relative humidity at about 30–34 °C. Active drinking and expectoration of secretions should be encouraged. Possible complications related to NPPV (e.g., poor NPPV tolerance, asynchrony due to leaks, nasal/facial skin necrosis, conjunctivitis, sinus/ear pain, nasal/oral dryness, gastric distention, aspiration, hypotension, acute respiratory distress syndrome, pneumothorax, and claustrophobia) are systematically evaluated and alleviated as much as possible.

### NPPV weaning and failure

For all patients, the levels of IPAP and EPAP and the daily use of NPPV can be gradually decreased under conditions of clinical stability, defined as the presence of the following: the improvement or resolution of the underlying cause of AECOPD, RR < 25 breaths/min, heart rate < 110 beats/min, arterial pH < 7.35, and PaO_2_ > 60 mmHg at FiO_2_ ≤ 0.4. Attempts to withdraw NPPV are made if IPAP is decreased to 10–12 cmH_2_O, EPAP is 5 cmH_2_O, or daily use is less than 5 h. Weaning is considered successful if, for 72 h after withdrawal, patients are able to sustain spontaneous breathing without signs of respiratory distress, defined as the presence of at least two of the following: arterial pH < 7.35; RR > 30 breaths/min; PaO_2_ < 60 mmHg or arterial oxygen saturation measured by pulse oximetry (SpO_2_) < 90% at FiO_2_ ≥ 0.4; retraction of the intercostal spaces, use of accessory respiratory muscles, or thoracic–abdominal paradoxical movement; or decreased consciousness, agitation, or diaphoresis [30]. By contrast, when patients present with the aforementioned signs of respiratory distress after withdrawal, weaning is considered to have failed and NPPV is resumed.

Endotracheal intubation is considered if a patient has either arterial pH < 7.25 with PaCO_2_ > 20% greater than the baseline or PaO_2_ < 60 mmHg despite maximum tolerated supplemental oxygen, and if at least one of the following criteria is met: clinical signs suggestive of severely decreased consciousness (e.g., coma, delirium); excessive amount of respiratory secretions with weak cough; use of accessory respiratory muscles or thoracic–abdominal paradoxical movement; severe upper gastrointestinal bleeding with aspiration or vomiting; or severe hemodynamic instability, despite fluid repletion and use of vasoactive agents [29]. If a patient fulfills one of these criteria, the final decision for intubation is made by the attending physician, with the consent of the patient’s next of kin or other surrogate decision makers, as appropriate.

### General therapy for AECOPD

All participating patients, regardless of the study arm into which they are randomized, will be monitored and managed following the recommendations of the 2017 GOLD guidelines [1] and according to each center’s specific expertise and clinical routine.

The following approaches should be considered (but are not mandatory) for the use of bronchodilators: increasing doses and/or frequencies of short-acting bronchodilators, if required; the combination of short-acting beta2-agonists and anticholinergics, if required; the use of long-acting bronchodilators, if the patient becomes stable; and the use of spacers or air-driven nebulizers, when appropriate. Corticosteroids (oral/intravenous) should be considered if needed. Antibiotics (oral/intravenous) are considered when signs of bacterial infection are present. The specific drugs used and the dosing regimens will be left up to the attending physician.

At all times, the following procedures should be followed: monitoring fluid balance, considering subcutaneous heparin or low molecular weight heparin for thromboembolism prophylaxis, and identifying and treating associated conditions (e.g., heart failure, arrhythmias, and pulmonary embolism).

The study protocol requires that routine monitoring include measurements of noninvasive blood pressure, pulse oximetry, and electrocardiography. Every patient should receive at least one peripheral venous line to receive intravenous treatments during the study period. Nasogastric tubes, urinary bladder catheters, and/or other intravenous catheters may be used at the discretion of the attending physician, following established protocols at each center. Any decision affecting the protocol will be recorded in the case report form (CRF).

### Data collection and definitions

At each study center, patient data will be collected using that center’s dedicated pseudonymous CRF, and the CRFs will be stored in locked file cabinets in areas with limited access. Data will be transmitted to the coordinating center whenever a patient dies or is discharged from the hospital, and follow-up data will be transmitted to the coordinating center when follow-up is complete. In addition, the coordinating center will have direct access to the cloud platform to extract the data related to the NPPV parameters. Before the data are exported into a computerized database at the coordinating center, two trained data collectors from the coordinating center will check the completeness and quality of information. Logical checks will be performed for missing data and to determine inconsistencies. If required, the data collector will contact the investigator by phone to validate or reformat the data for entry into the database. Then all data entry will be performed separately by two trained data collectors, and their work will be checked by another two collectors. Only the principal investigator and the data managers at the coordinating center will have access to the final database.

At enrollment, patients’ baseline characteristics will be recorded: demographics, medical history, history of NPPV use, history of steroids and/or inhalation therapy use, smoking status, spirometry (forced ventilatory capacity and forced expiratory volume in 1 s), Acute Physiology And Chronic Health Evaluation (APACHE) II score, vital signs (including heart rate, RR, arterial blood pressure, SpO_2_), dyspnea score, scale for the use of accessory muscles, Glasgow coma scale (GCS) score, arterial blood gases (including pH, PaO_2_, PaCO_2_, bicarbonates, and lactates), and routine laboratory tests (including hemoglobin, white blood cell count, platelet count, prothrombin time, partial thromboplastin time, creatinine, blood urea nitrogen, alanine aminotransferase, aspartate aminotransferase, and bilirubin).

At 2, 6, 24, and 48 h after randomization, vital signs, dyspnea score, scale for the use of accessory muscles, GCS score, supplemental oxygen flow, and arterial blood gases will be recorded.

All NPPV parameters will be continuously monitored by the built-in pneumotachograph and uploaded to the cloud platform, which will provide data continuously over a wireless network. Peak inspiratory pressure (PIP), EPAP, exhaled *V*_T_, frequency, exhaled minute volume (*V*_m_), and leak at 2, 6, 24, and 48 h after randomization will be extracted from the cloud platform and recorded with those averaged values over 5 min at the indicated time points. In addition, maximal PIP, maximal EPAP, daily time of NPPV use (hours) over the first 5 days, and total time of NPPV use (days and hours) will be recorded by the cloud platform. Complications related to NPPV will be monitored and recorded.

NPPV tolerance will be recorded on a 4-point scale and then dichotomized into acceptable (score of 2 or 3) or poor (score of 0 or 1) tolerance [31]. The dyspnea score will be assessed using a verbal analog scale with scores from 0 (no dyspnea) to 10 (maximum dyspnea). The use of accessory muscles will be assessed according to the following scale: 0, no visible tonic or phasic use of neck muscles; 1, neck muscles taut but with no respiratory modulation (i.e., tonic activity); 2, mild respiratory modulation in neck muscle contraction; 3, moderate phasic activity (no supraclavicular or intercostal indrawing); 4, vigorous phasic activity with indrawing; and 5, vigorous phasic activity with abdominal paradox [32]. To determine PaO_2_/FiO_2_ ratios while patients are receiving NPPV, FiO_2_ will be calculated using the following conversion factor: (21% + [3% × oxygen flow in L/min of supplemental oxygen]). This provides an approximation of percent oxygen delivered, is influenced by minute ventilation and breathing patterns, and may be inaccurate if air leakage occurs around the mask or through the mouth [33].

### Endpoints and follow-up

The primary endpoint is the NPPV failure rate, defined as the need for endotracheal intubation and invasive ventilation at any point during the study. Secondary endpoints include decrement of PaCO_2_ from baseline to 2 h after randomization, hospital mortality, 28-day mortality, and 90-day survival, as well as actual intubation rate, intensive care unit (ICU) admission, hospital stay, hospital costs, ventilator-free days within 28 days, hospital-free days within 90 days, and 90-day hospital readmission.

All patients will be monitored until hospital discharge or until day 90 in cases where the patients remain at the hospital. After discharge, follow-up will be performed by phone on days 28 and 90 after randomization, and the following will be recorded: 28-day mortality, ventilator-free days within 28 days, hospital-free days within 90 days, 90-day survival, and 90-day hospital readmission.

### Study dropouts

Because participation is voluntary, patients have the right to withdraw consent to participate at any time for any reason, without any unfavorable consequences for further medical treatment. Furthermore, investigators have the right to terminate the participation of any patient at any time if the investigator deems it to be in the participant’s best interest. The reasons and circumstances for study discontinuation will be documented in the CRF.

### Sample-size calculations and interim analyses

The calculation of the sample size was based on our primary hypothesis that in AECOPD patients high-intensity NPPV leads to a reduced need for endotracheal intubation compared to low-intensity NPPV. Our previous clinical experience and previous studies [18–20] led us to anticipate that the need for endotracheal intubation would be 15% for the low-intensity NPPV group. Based on the assumption that the need for intubation could be reduced to 6% in the high-intensity NPPV group, we estimated that a minimum sample size of 480 patients would be required to detect a between-group difference of 9% for the need for intubation, with an 81% power and a two-tailed alpha level of 0.05, using superiority tests for two proportions. Assuming a dropout rate of 20%, we calculated that 600 patients should be enrolled.

Interim analysis to assess superiority is scheduled, and it will take place at hospital discharge of the first 300 randomized patients. The criteria for premature discontinuation of the study, based on the results of this single interim analysis, will be those defined by O’Brien and Fleming [34]. It will be performed at the coordinating center by an independent data and safety monitoring board (DSMB). The DSMB will meet by conference call, and DSMB discussions will take place by email or conference call.

### Statistical analysis

We will report continuous variables as means ± standard deviations or medians and interquartile ranges, when appropriate, and categorical variables as proportions.

Continuous variables will be compared using an appropriate parametric (Student’s *t* test) or nonparametric (Mann–Whitney *U* test) method. Categorical variables will be compared using a chi-square test or Fisher’s exact test, if appropriate. A linear mixed model with two factors (study group and time) will be used to analyze variables repetitively measured over time; Bonferroni’s adjustment for multiplicity of tests will be used in post hoc comparisons to ensure that the total error rate will not exceed 0.05.

We will use a chi-square test or Fisher’s exact test to compare the primary outcome and secondary dichotomous outcomes, and a parametric (Student’s *t* test) or nonparametric (Mann–Whitney *U* test) method to compare secondary continuous outcomes, as appropriate. For the primary and secondary outcomes, we will calculate between-group differences in risk for dichotomous outcomes, between-group median differences (using Hodges–Lehmann estimates) for continuous outcomes with non-normal distributions, and between-group mean differences for continuous outcomes with normal distributions, all with 95% confidence intervals. The baseline characteristics will be tested for imbalance. If imbalances are found (despite the 1:1 randomization of a relatively large cohort), the primary and secondary outcome variables will be analyzed using a multiple logistic regression model, adjusted for possible baseline imbalances.

Cumulative event rates for the primary and secondary endpoints involving time-to-event data will be estimated for the two groups using the Kaplan–Meier method and compared using a log-rank test. Hazard ratios, their 95% confidence intervals, and *P* values for the comparison of the two treatment groups will be determined using the Cox regression model.

In case of loss to follow-up or consent withdrawal, the causes will be reported. Intention-to-treat (ITT) and per protocol (PP) analyses will be conducted. For the ITT analysis, data will be processed for all trial patients in the groups in which they were randomized. A PP analysis will be performed as a sensitivity analysis in the case of any loss to follow-up, a missing primary endpoint, or any protocol deviation that might lead to a biased conclusion. Missing data will be handled using the “last observation carried forward” method.

All tests will be two-sided. Differences with *P* values of less than 0.05 will be considered statistically significant, except for those incorporating multiple comparisons. Statistical analyses will be performed using SPSS software (version 19.0, SPSS Inc., Chicago, IL, USA).

### Trial organization

Appendix 1 lists the investigators of the HAPPEN collaboration group. The steering committee is composed of the principal investigators in the study, who all contributed to its design and approved the final protocol (Appendix 2). The executive committee is made up of the main investigators at each participating center and is responsible for administrative, trial, and data management (Appendix 2). When the report on the results of the trial is submitted for possible publication, each study center will be eligible for one co-authorship, plus a further co-authorship for every 20 patients with complete datasets.

The DSMB is composed of three external, independent experts in critical care medicine, NPPV, and COPD (Appendix 2). At the interim analysis, using the general data provided by the three internal members, it will recommend the continuation or discontinuation of the trial, based on the available data from the interim analysis. The DSMB will be responsible for monitoring patient data and safety, including review of the protocol, with emphasis on data integrity and participant risk and safety issues, monitoring adverse events (including suspected severe adverse reactions), and monitoring the overall status of the study (e.g., progress of patient enrollment, general adherence to protocol, and completeness of data entry).

The trial management team includes a chief investigator, a project manager, a statistician, a clinical epidemiologist, and an investigator expert in clinical trials (Appendix 2). The responsibilities of this team are as follows:

1. Planning and conducting the study: designing the protocol and CRF, designing the investigator manual, and managing and controlling data quality
2. Research center support: assisting the centers with administrative submission, monitoring recruitment rates, providing sealed randomization envelopes, taking actions to increase patient enrollment, monitoring follow-up, auditing, and sending study materials to the research centers
3. Producing a monthly study newsletter and developing supporting material for the study
4. Programming a research-in-progress meeting at least once every 6 months with the principal investigators from all sites
5. Statistical analysis and research reporting: interim and complete statistical analysis and helping to write the final manuscript.

## Discussion

NPPV is increasingly used in the care of patients with AECOPD, and several lines of evidence strongly support its use in these patients [7–19]. However, it still fails in approximately 15% of AECOPD patients; in these cases, mortality is not reduced [18–20]. Plant et al. [19] conducted a randomized controlled trial that indicated a 15% failure rate of NPPV in patients with mild to moderate respiratory acidosis in the general ward setting. In addition, Contou et al. [20] performed an observational cohort study in an experienced ICU and reported an NPPV failure rate of 15% in COPD patients with acute hypercapnic respiratory failure. In addition, a meta-analysis by Ram et al. [18] showed that the intubation rate was 16.4% in such patients when they received NPPV.

There are four possible ways to explain NPPV failure in the conventional approach, i.e., using low-intensity NPPV. The main reason for NPPV failure is that, despite NPPV use, pH and PaCO_2_ continuously worsen in conventional low-intensity NPPV, and then consciousness may be difficult to recover in a small percentage of AECOPD patients [20–22]. Another reason is that, despite significant improvement in ventilatory status, PaCO_2_ is difficult to normalize and can easily increase when there is a slight change in a patient’s clinical situation, which often triggers NPPV failure [16, 19]. Moreover, it remains to be seen whether continuously elevated PaCO_2_ is harmful to the internal environment or vital organs. Further, a minority of patients with AECOPD tolerate conventional NPPV poorly, to the point where it is discontinued, possibly because of inadequate pressure support provided by low-intensity NPPV. Ultimately, in such cases, endotracheal intubation is required [23, 24]. Thus, enhancing the pressure-support intensity of NPPV might be of critical importance to reduce the need for intubation, in turn reducing the mortality rate.

High-intensity NPPV is a novel therapeutic option which can be used to maximally decrease severely elevated PaCO_2_ to normal levels [25, 26]. In theory, high-intensity NPPV may be more efficient than low-intensity NPPV for augmenting alveolar ventilation and offsetting the extra dead space caused by the face mask, and reducing the inspiratory work of breathing and alleviating dyspnea in a way that provides more comfort during NPPV. Windisch et al. [27] reported that, in patients with stable hypercapnic COPD, improvements in PaCO_2_ levels, lung function, and breathing pattern were achieved using high-intensity NPPV. Dreher et al. [28] also directly compared high-intensity NPPV with the conventional approach of low-intensity NPPV in patients with stable hypercapnic COPD, and found that high-intensity NPPV was superior in terms of controlling nocturnal hypoventilation, thus improving dyspnea during physical activity, lung function, and health-related quality of life. However, whether high-intensity NPPV is superior to low-intensity NPPV in the treatment of AECOPD patients remains unclear. Hence, our aim is to verify whether high-intensity NPPV is more effective for correcting hypercapnia than low-intensity NPPV, thereby reducing the need for intubation and improving survival in AECOPD patients.

For low-intensity NPPV, we opted to adjust IPAP up to 20 cmH_2_O, according to patients’ tolerance, to obtain a possible target *V*_T_ of 10 mL/kg, regardless of whether normocapnia is achieved, a level confirmed by the literature and our routine clinical practice [13, 19, 29]. For high-intensity NPPV, we opted to adjust IPAP up to 30 cmH_2_O according to patients’ tolerance, to obtain a possible target *V*_T_ of 15 mL/kg [25, 26]. Importantly, the major target for the adjustment of IPAP is achieving normocapnia as far as possible [25]. The target *V*_T_ of 15 mL/kg is set to offset the extra dead space caused by the face mask over the *V*_T_ of 10 mL/kg in the low-intensity NPPV group, and precisely this level was chosen because the inner volume of the face mask is about 240–375 mL, which is approximately equal to 5 mL/kg [35]. To prevent ventilator-induced lung injury, we have set the upper limit for IPAP to 30 cmH_2_O, which is commonly considered safe [36].

The HAPPEN study protocol has limitations which must be addressed. First, the study is being performed in patients with mild to moderate respiratory acidosis, which suggests that the results may not be generalizable to the whole population. Further study will be required to assess whether our findings can be extended to other subgroups and the whole population. Second, despite the blinding of patients to the intervention, blinding of investigators is not possible due to the nature of the intervention, and this could induce bias. However, at each study center, at least two investigators will be working. One investigator will perform the interventions, as defined in the protocol. The second investigator, who will be blinded to the intervention, will collect data. Moreover, all data related with the parameters of NPPV will be extracted, and all data analyses will be performed in a blinded fashion. Third, even if protocol interventions represent a consensus among centers, between-center differences in the experience of NPPV use may still affect the results of this study. Fourth, several confounding factors associated with general therapy for AECOPD (e.g., the use of bronchodilators, corticosteroids, and antibiotics; fluid administration; thromboembolism prophylaxis; treatment of associated conditions) are only suggested and are not protocolized. Nevertheless, the study protocol stresses that general therapy is to be performed in accordance with each center’s specific expertise to ensure that the trial is as close as possible to routine clinical care.

In summary, the HAPPEN study will be the first randomized controlled study to investigate whether high-intensity NPPV better corrects hypercapnia and reduces the need for intubation and mortality in AECOPD patients than low-intensity NPPV. The results of the HAPPEN study will help critical care physicians choose the intensity at which to deliver NPPV to AECOPD patients.

## Trial status

The HAPPEN study is currently recruiting patients. Recruitment began in December 2017. The expected duration of the study is 12 months. The final results will be published as soon as possible after the analysis is completed.

### Additional files

Additional file 1: SPIRIT 2013 checklist: recommended items to address in a clinical trial protocol and related documents. (DOC 122 kb)

Additional file 2: Informed consent form. (DOC 43 kb)

## Appendix 1

Investigators of the HAPPEN collaboration group (in alphabetical order by hospital):

Lianxiang Guo and Xiaohua Hou (The Affiliated Hospital of Henan Polytechnic University, Jiaozuo, China); Yunhui Lv and Rui Sun (The Affiliated Hospital of Kunming University of Science and Technology, Kunming, China); Chen Liu and Panpan Zhang (The Affiliated Hospital of North China University of Science of Technology, Tangshan, China); Zhixin Cao, Hangyong He, Qian Li, Sijie Liu, Zujin Luo, Yingmin Ma, Baosen Pang, Bing Sun, Zhaohui Tong, and Jian Zhu (Beijing Chao-Yang Hospital, Capital Medical University, Beijing, China); Fucheng An and Pengfei Li (Beijing Mentougou District Hospital, Beijing, China); Xiuhong Jing and Hongru Liu (Beijing Pinggu Hospital, Beijing, China); Chao Zhang and Yongxiang Zhang (Beijing Renhe Hospital, Beijing, China); Mingdong Hu and Qi Li (Chongqing Xin-Qiao Hospital, Army Military Medical University, Chongqing, China); Zhijun Feng and Wujie Lu (The First Affiliated Hospital of Henan University, Kaifeng, China); Mingwei Chen and Tianjun Chen (The First Affiliated Hospital of Xi’an Jiaotong University, Xi’an, China); Shengyu Wang and Jing Zhou (The First Affiliated Hospital of Xi’an Medical University, Xi’an, China); Yaoming Hu and Manyan Zhang (The First Hospital of University of South China, Hengyang, China); Wenshuai Feng and Lujiang Li (Gongyi City People’s Hospital, Gongyi, China); Hongmei Wu and Ling Yu (Haicheng Central Hospital, Haicheng, China); Yongpeng An and Xiuzhi Yang (Kaifeng Central Hospital, Kaifeng, China); Qiang Dang and Dawei Zheng (Nanyang Central Hospital, Nanyang, China); Yonghong Xiang and Yurong Zhang (National Hospital of Guangxi Zhuang Autonomous Region, Nanning, China); Xiuhong Ma and Zhiyang Yin (People’s Hospital of Beijing Huairou District, Beijing, China); Dan Wang and Longguang Wu (People’s Hospital of Hanshou, Hanshou, China); Chao Wu and Xiaohong Yang (People’s Hospital of Xinjiang Uygur Autonomous Region, Urumqi, China); Dongmei Li and Heli Wang (Sanmenxia Central Hospital, Sanmenxia, China); Runxia Shao and Xiaoping Zhang (The Second Affiliated Hospital of Zhengzhou University, Zhengzhou, China); Rongbo Wang and Xinghui Wu (The Second Hospital of Baoji, Baoji, China); Lin Chen and Xiaowu Tan (The Second Hospital of University of South China, Hengyang, China); Kailiang Duan and Huiqing Ge (Sir Run Run Shaw Hospital, Zhejiang University School of Medicine, Hangzhou, China); Zhifang Liu and Sanhong Zhang (The Third Hospital of Baoji, Baoji, China); and Juan Wang and Kailv Wang (The Third Hospital of Mianyang, Mianyang, China).

## Appendix 2

Steering committee (in alphabetical order by investigator): Zhixin Cao, Huiqing Ge, Qi Li, Yingmin Ma, Baosen Pang, Bing Sun, Zhaohui Tong, and Xiaohong Yang.

Executive committee (in alphabetical order by investigator): Fucheng An, Yongpeng An, Lin Chen, Mingwei Chen, Qiang Dang, Kailiang Duan, Wenshuai Feng, Zhijun Feng, Lianxiang Guo, Mingdong Hu, Yaoming Hu, Dongmei Li, Chen Liu, Hongru Liu, Zhifang Liu, Zujin Luo, Yunhui Lv, Xiuhong Ma, Runxia Shao, Kailv Wang, Shengyu Wang, Chao Wu, Longguang Wu, Xinghui Wu, Yonghong Xiang, Ling Yu, and Yongxiang Zhang.

Data and safety monitoring board (in alphabetical order by investigator): Bing Dai, Lixin Xie, and Qingyuan Zhan (external members); Sijie Liu, Zujin Luo, and Jian Zhu (internal members).

Trial management team (in alphabetical order by investigator): Zhixin Cao, Hangyong He, Lirong Liang, Yichong Li, and Zujin Luo.

## Abbreviations

AECOPD: Acute exacerbation of chronic obstructive pulmonary disease
CONSORT: Consolidated Standards of Reporting Trials
COPD: Chronic obstructive pulmonary disease
CRF: Case report form
DSMB: Data and safety monitoring board
EPAP: Expiratory positive airway pressure
FiO_2_: Fraction of inspired oxygen
GCS: Glasgow coma scale
GOLD: Global Initiative for Chronic Obstructive Lung Disease
HAPPEN: High-intensity versus low-intensity noninvasive positive pressure ventilation in patients with acute exacerbation of chronic obstructive pulmonary disease
ICU: Intensive care unit
IPAP: Inspiratory positive airway pressure
ITT: Intention to treat
NPPV: Noninvasive positive pressure ventilation
PaCO_2_: Arterial carbon dioxide tension
PaO_2_: Arterial oxygen tension
PBW: Predicted body weight
PIP: Peak inspiratory pressure
PP: Per protocol
RR: Respiratory rate
SPIRIT: Standard Protocol Items: Recommendations for Interventional Trials
*V*_m_: Minute volume
*V*_T_: Tidal volume
