# Supplementary material for: High-intensity versus low-intensity noninvasive positive pressure ventilation in patients with acute exacerbation of chronic obstructive pulmonary disease (HAPPEN): study protocol for a multicenter randomized controlled trial
Source: Trials. 2018 Nov 21;19:645. doi: 10.1186/s13063-018-2991-y (PMC6249746; doi:10.1186/s13063-018-2991-y)
Supplement: Supplementary file 2 — Informed consent form. (DOC 43 kb) [file 13063_2018_2991_MOESM2_ESM.doc]

**Informed Consent Form**

**Study title:** **High-intensity versus low-intensity noninvasive positive pressure ventilation in patients with acute exacerbation of chronic obstructive pulmonary disease (HAPPEN): study protocol for a multicenter randomized controlled trial**

**Principal investigator:** Zhixin Cao

**Study organization:** The HAPPEN collaboration group

**Sponsor:** Beijing Chao-Yang Hospital

**Introduction**

I am an investigator working for the HAPPEN collaboration group. We are performing a randomized controlled study on high-intensity versus low-intensity noninvasive positive pressure ventilation in patients with acute exacerbation of chronic obstructive pulmonary disease (AECOPD). This study is recruiting 600 AECOPD patients with low-to-moderate hypercapnic respiratory failure admitted to a network of 27 respiratory wards from university hospitals in China. The final protocol has been approved by the ethics committee at each participating hospital.

I will provide information to you and invite you to be part of this study. You do not have to decide today whether you will participate. Before you decide, you can talk to anyone you feel comfortable with about this decision. There may be some words that you do not understand. Please ask me to stop if you do not understand anything as we go through the information, and I will take the time to explain it. If you have questions later, you can ask them of me or the associated staff.

**Purpose**

AECOPD is defined as an acute worsening of respiratory symptoms that results in the need of additional therapy, and it is characterized as an acute clinical event that negatively impacts health status and hospitalization and readmission rates, even possibly increasing rates of comorbidities and COPD-related mortality. Over the past two decades, noninvasive positive pressure ventilation (NPPV) has been increasingly used in the care of AECOPD patients. Several lines of evidence strongly support its use in such patients, including a large number of favorable findings that NPPV, over conventional oxygen therapy, corrects ventilatory status, reduces the need for invasive ventilation, and improves prognosis. However, in some AECOPD patients, NPPV fails and invasive ventilation is required; in such cases, the mortality rate may not be reduced. To rectify this, we are performing a randomized controlled study, which intends to assess the efficacy of high-intensity NPPV in the care of AECOPD patients compared to low-intensity NPPV. The basic principle of this study is to guarantee you favorable diagnosis and treatment, and all data we will collect about you will be used for scientific research and better treatment for you and other AECOPD patients.

**Please read the following explanation carefully:**

1. All measures related to diagnoses and treatments are taken according to your clinical condition; there are no special measures arranged for the study.

2. For this study, we invited many well-known clinical experts to establish treatment protocol for participants to give you the most correct diagnosis and treatment and minimize possible risks. Moreover, we will choose the most appropriate treatment program with reference to your clinical condition.

3. The treatment protocol used in this study has been confirmed by research to be effective for improving your clinical condition. In theory, high-intensity NPPV might correct hypercapnia better, reducing the need for intubation and the mortality rate for patients like you. There may not be any benefits for you, but your participation is likely to help us find the answer to this study question.

4. As with any other treatment, the treatments used in this study could possibly be accompanied by adverse events (e.g., pneumothorax, abdominal distension). We will certainly explain the possible adverse events to you in advance and minimize possible risks. If such an adverse event happens, we will do our best to reduce any related injuries as much as possible.

5. There is no extra cost for inclusion in this study, nor will you be given any money or gifts to take part in this study. If an adverse event occurs, all relevant medical costs will be paid by us.

6. Both the information gathered from you and the results of the research will be treated confidentially. The data obtained will only be published in an anonymous and aggregated way. Unless you have provided specific authorization, your personal results will not be made available to any third parties.

7. Because participation in the study is voluntary, you have the right to withdraw consent at any time for any reason, without any unfavorable consequences regarding further treatment. At any stage of this study, you can ask questions, and these will be promptly answered by your physicians. Should you wish to withdraw your data from the database, you will be free to do so without further explanation. Furthermore, the investigators have the right to terminate your participation at any time if the investigator deems it to be in your best interest.

8. If you have any questions, you may ask them now or later, even after the beginning of the study. If you wish to ask questions later, you may contact Dr Zujin Luo by telephone (phone number: 13810497587).

**Certificate of Consent**

**Statement by the participant**

I have read the foregoing information or it has been read to me. I have had the opportunity to ask questions about it, and all questions that I have asked have been answered to my satisfaction. I fully understand the purpose, content, benefits, and possible adverse events of this study. I have been informed that participation in the study is voluntary, I can ask questions of the physicians at any time, and I have the right to withdraw consent to participate in the study at any time for any reason, without any unfavorable consequences for further treatment.

**I consent to voluntary participation in this study.**

**Participant signature**

Print Name Signature Date

**Signature of surrogate decision maker**

Print Name Signature Date______________

Relationship with the participant

**Statement by the investigator**

I have accurately read out loud the information sheet to the potential participant, and, to the best of my ability, made sure that the participant (or the surrogate decision maker) understands the purpose, content, benefits, and possible adverse events of this study. I confirm that the participant was given an opportunity to ask questions about the study, and all questions asked by the participant have been answered correctly and to the best of my ability. I confirm that the participant has not been coerced into giving consent, and consent has been given freely and voluntarily.

 A copy of this informed consent form has been provided to the participant.

**Investigator signature**

Print Name ________________ Signature________________ Date________________

**知情同意书 (Chinese Version)**

**项目名称：**高强度无创正压通气治疗慢性阻塞性肺疾病急性加重的前瞻性多中心随机对照研究

**项目负责人：**曹志新

**研究机构：**高强度无创通气治疗慢性阻塞性肺疾病多中心研究协作组

**牵头单位：**首都医科大学附属北京朝阳医院

**简要介绍**

我是高强度无创通气治疗慢性阻塞性肺疾病多中心研究协作组的一名研究者。我们正在开展高强度无创正压通气治疗慢性阻塞性肺疾病的多中心研究。这项研究预计在国内27家医院纳入600例患者，并已通过各家单位伦理委员会的审查和批准。我将向您提供研究的相关信息并邀请您参加这项研究。您可以考虑，没有必要今天给出明确答案。在您决定是否参加之前，您可以咨询您认为合适的任何人。这里可能有些术语您不太理解，在与您沟通时您可以随时向我提问，我将尽我所能为您解释。在这之后如有问题，您也可以向我、管床医生及其他工作人员咨询。

**研究目的**

慢性阻塞性肺病急性加重（AECOPD）是临床常见的呼吸重症，是导致COPD反复住院、致残、致死、加重疾病负担的主要原因。近年来，无创正压通气NPPV广泛应用于AECOPD患者，临床研究表明与常规治疗相比，NPPV可明显降低患者气管插管率及病死率，可显著改善患者预后，但仍有较高比例的患者面临NPPV失败，需要气管插管并接受有创通气，导致这部分患者病死率居高不下。为了攻克这一医学难题，本单位正在进行一项关于降低AECOPD气管插管率及病死率的研究，其目的在于验证高强度NPPV降低AECOPD气管插管率和病死率的有效性及安全性。本课题组以您得到及时、正确的诊断和治疗为主要目的，所取得的临床资料将用于您和其他AECOPD患者的治疗和科学研究。

**我们就以下内容向您说明，请您仔细阅读：**

1、对您的诊断与治疗均是根据病情需要安排的，没有任何仅以研究为目的项目。

2、本研究由国内在本领域知名的专家为患者制定合理的治疗方案，使您获得规范诊治，同时将风险减至最低。在整个研究过程中，我们将根据您的病情和医疗需要，选择适宜的治疗方案，为您提供完善的医疗服务。

3、本研究采用的治疗方案，经过大量基础与临床研究证实有一定疗效，对您的病情应该是有益的。理论上讲，高强度NPPV更能改善您的通气效果，降低气管插管率以及改善您的临床预后。当然，研究结果可能对您没有任何益处，但是您的参与对于帮助我们回答该科学问题仍然具有重要价值。

4、但与任何治疗一样，本研究可能会产生某些不良反应，如气压伤、腹胀等。对于可能发生的情况，我们事先会如实地向您说明，并尽力将不良反应的发生机率降至最低。一旦出现某些不良反应，我们会及时采取有效措施，最大程度地减少损伤。

5、参与本研究不会增加不必要的医疗费用；参与本研究您不会得到任何报酬；如果患者出现与研究相关的任何不良反应，我们将承担由此造成的全部费用。

6、您的资料只有相关的医生和研究人员知道。未经您的允许，我们不会把这些资料提供给他人。

7、参与本研究完全是自愿的，在研究期间您和您的家属均有权提出随时退出研究，但不影响今后对患者的进一步治疗。在研究过程中的任何时候，您及您的家属均可询问关于病情的任何问题，医生会及时给予解答。您可以随时从数据库内撤离您的相关信息，并不需要任何解释。若您由于某些特殊原因不适合参加本研究，医生也有权终止您参与研究。但无论因何种原因终止研究，我们仍将尽最大所能为患者提供规范化的诊断与治疗，同时也请您或您的家属继续配合医生治疗。

8、您在研究期间有任何问题，都可以向相关工作人员询问，或者可以电话联系罗祖金医生进行电话咨询（电话号码：13810497587）。

**知情同意签署**

**患者声明**

我已经仔细阅读了上述声明，或者是研究人员向我进行了仔细讲解。我已经获得了我问问题的机会，并且我问的问题都得到了圆满的回答。我已清楚地了解本研究的目的、内容、好处以及可能发生的不良反应。我知道我参加本项研究是完全自愿的，可以随时提出任何问题，并能够得到医生的详细解答，以及可以在任何时候退出研究，而不会导致任何不良后果。

**我已经阅读了上述声明并同意参加本项研究。**

**患者签名**

打印体______________手写体 ______________ 日期 ______________

**或法定代理人签名**

打印体______________手写体 ______________ 日期 ______________

与患者关系______________

**研究者声明**

我已经向患者仔细阅读了相关信息，并尽我所能确保患者或法定代理人理解本研究的目的、内容、好处以及可能发生的不良反应。我保证患者已经得到了提问的权利，并已尽我所能正确地回答了患者所提出的所有问题。我保证在签署知情同意书时患者没有遭到任何胁迫，且在自由、自愿的情况下签署的。

该知情同意书的1份复印件已经提供给患者。

**研究者签名**

打印体______________ 手写体 ______________ 日期 _____________
